# Preoperative plasma visfatin may have a dual effect on the occurrence of postoperative delirium

**DOI:** 10.3389/fmed.2022.1024942

**Published:** 2022-11-22

**Authors:** Ning Kang, Ning Yang, Kaixuan Zhao, Zhengqian Li, Wenchao Zhang, Yongzheng Han, Kaixi Liu, Yanan Song, Lei Chen, Yue Li, Jingshu Hong, Yitong Li, Xiangyang Guo, Geng Wang, Yi Yuan

**Affiliations:** ^1^Department of Anesthesiology, Peking University Third Hospital, Beijing, China; ^2^Department of Anesthesiology, Beijing Jishuitan Hospital, Beijing, China; ^3^Beijing Center of Quality Control and Improvement on Clinical Anesthesia, Beijing, China

**Keywords:** hip fracture, visfatin, postoperative delirium, interleukin-6, mediation analysis

## Abstract

**Background:**

Visfatin is considered to be a “novel pro-inflammatory cytokine.” Neuroinflammatory response is one of the important mechanisms of postoperative delirium (POD). The relationship between preoperative plasma visfatin and POD is unclear.

**Objective:**

To investigate the relationship between preoperative plasma visfatin concentrations and POD (primary outcome) in older hip fracture patients and to explore whether it affects POD through inflammatory factors.

**Materials and methods:**

This prospective cohort study enrolled 176 elderly patients who were scheduled for hip fracture surgery. Preoperative plasma was collected on the morning of surgery, and visfatin levels were measured. Interleukin (IL)-1 and IL-6 were measured using patients’ plasma collected on the first day after surgery. We used the 3-min diagnostic interview for Confusion Assessment Method-defined delirium (3D-CAM) twice daily within the 2 days after surgery to assess whether POD had occurred. Restricted cubic splines and piecewise regression were used to explore the relationship between preoperative plasma visfatin concentrations and POD, and further mediation analysis was used to verify whether visfatin plays a role in POD through regulating inflammatory factors.

**Results:**

The incidence of POD was 18.2%. A J-shaped association was observed between preoperative plasma visfatin levels and POD. The risk of POD decreased within the lower visfatin concentration range up to 37.87 ng/ml, with a hazard ratio of 0.59 per 5 ng/ml [odds ratio (OR) = 0.59, 95% confidence interval (CI) = 0.37–0.95], but the risk increased above this concentration (*P* for non-linearity < 0.001, with a hazard ratio of 1.116 per 10 ng/ml; OR = 1.10, 95% CI = 1.02–1.23). Mediation effect analysis showed that when the plasma visfatin concentration was higher than 37.87 ng/ml, the effect of visfatin on POD was mediated by IL-6 (*p* < 0.01). A significant indirect association with postoperative plasma IL-6 was observed between preoperative plasma visfatin and POD (adjusted β = 0.1%; 95% CI = 4.8∼38.9%; *p* < 0.01).

**Conclusion:**

Visfatin is the protective factor in POD when the preoperative plasma visfatin concentration is below 37.87 ng/ml, but when it exceeds 37.87 ng/ml, the visfatin concentration is a risk factor for POD, which is mediated by postoperative plasma IL-6. The results suggest that preoperative visfatin may have a dual effect on the POD occurrence.

**Clinical trial registration:**

[www.ClinicalTrials.gov], identifier [ChiCTR21 00052674].

## Introduction

Hip fracture is a main disease that threaten the health and quality of life in older adults, and its incidence increases with population aging. Epidemiological data showed that one in five women and one in ten men have hip fractures globally per year, and most of them need surgery (1). Older adults undergoing hip fracture surgery have high disability and mortality rates, which become a serious medical, economic, and social problem (2).

Postoperative delirium (POD) is a common complication after hip fracture surgery in older patients (3). It is mainly manifested by changes in the level of consciousness, cognitive dysfunction, decreased attention, and disturbance of the sleep-wake cycle. The incidence rate is approximately 4–61% (4), which is depending on the type of surgery. Although it can occur at any time during a patient’s hospital stay, POD usually develops during the first few days after surgery. POD is associated with poor outcomes such as longer duration of hospitalization, increased mortality rates, and increased morbidity (5). Additionally, it can affect the recovery of physical function and the return to normal life after discharge from the hospital.

The mechanism of POD mainly includes five theories including the neuroinflammation theory (6). A lot of studies have shown that IL-1 and IL-6 levels in the peripheral circulation are related to POD occurrence (7, 8).

Visfatin is a multi-functional protein molecule that is present in many organs and tissues in the body including brain (such as cortex and hippocampus area) and lung (9). Visfatin can exert multiple insulin simulation effects, including enhanced glucose intake and increased triglyceride synthesis (10). Visfatin is also regarded as a new type of inflammatory cytokine. Studies have shown that visfatin can activate monocytes to produce inflammatory cytokines such as interleukin (IL)-1ß, IL-6, and tumor necrosis factor α (TNF-α) (11). Recently, some studies showed increased concentrations of blood visfatin were associated with cognitive impairment (12–14). Moreover, some evidences which suggest that decreased visfatin levels might lead to dementia and Alzheimer’s disease (15, 16). However, some other studies appeared that visfatin played an important role in cognitive function. One study showed visfatin reduced hippocampal CA1 cell death and protects against memory loss and cognitive decline in male rats (17). Another study showed that mice lacking visfatin appearing hippocampal and cortical atrophy, astrogliosis, microgliosis, and abnormal CA1 dendritic morphology with altered intrahippocampal connectivity and abnormal behavior; including hyperactivity, some defects in motor skills and memory impairment (18). All these results above suggested that there may be a complex relationship between visfatin and cognitive function. Currently, the relationship between blood visfatin levels and POD is unclear. We suspected that there is also a complex link between visfatin and POD.

Therefore, we conducted this prospective cohort study to identify the correlation between preoperative plasma visfatin and POD and to further explore whether it can affect POD through inflammation factors.

## Materials and methods

### Patients and setting

**FIGURE 1:**
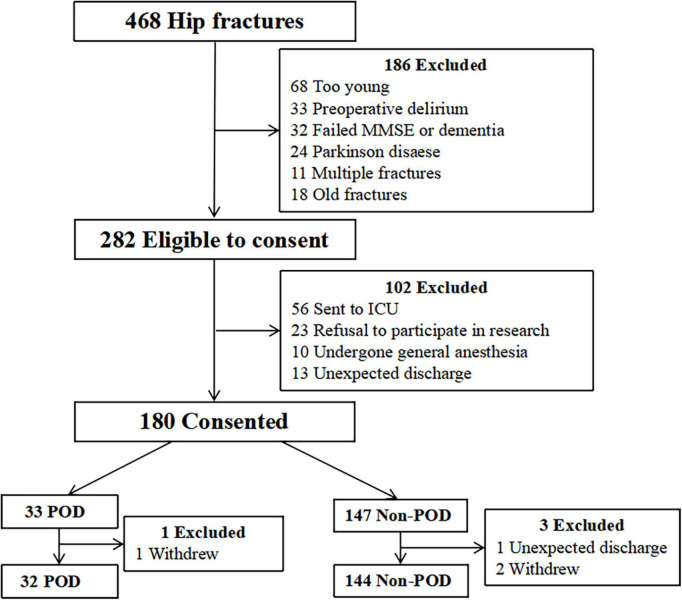
Study flow chart. Four hundred sixty-eight patients were initially screened for the study, and 176 patients were finally included in the data analysis. MMSE, mini-mental state examination; ICU, intensive care unit; POD, postoperative delirium.

This study was approved by the Beijing Jishuitan Hospital Medical Science Research Ethics Committee (JLKS202103-35) and was conducted in accordance with the principles of the Declaration of Helsinki. Cerebrospinal fluid (CSF) samples were obtained for the purpose of laboratory research. All methods were performed in accordance with relevant guidelines and regulations. Written informed consent was obtained, and this study was registered at the Chinese Clinical Trial Registry (ChiCTR2100052674). All participants were recruited from Beijing Jishuitan Hospital (Beijing, China) from November 2021 to April 2022. Eligible patients who were willing to participate in the study met the entry criteria described below. The inclusion criteria were as follows: at least 65 years old; diagnosed with unilateral hip fracture; operation 48 h after admission; hospital admission for surgical treatment of hip fracture; and American Society of Anesthesiologists (ASA) physical status classification II to III. There were 468 patients recruited into this study (Figure 1). All patients received intravenous patient-controlled analgesia after surgery (Sufentanil 0.1 μg/kg, flurbiprofen axetil 200 mg, tropisetron hydrochloride 10 mg, and normal saline to a volume of 100 ml). The exclusion criteria were as follows: dementia; psychiatric patients; preoperative delirium; Parkinson’s disease; preoperative mini-mental state examination (MMSE) scores was lower than 17 for illiterate, 20 for individuals with 1–6 years of education, 24 for individuals with seven or more years of education; nerve block contraindications (needle insertion site infection, local anesthetic allergy, coagulation dysfunction, international normalized ratio > 1.4, platelet count < 80 × 10^9^ μl); pathological fracture; metabolic bone disease; old fracture dementia; a stroke within 6 months; transferred to the intensive care unit (ICU) after surgery; difficulty communicating; and/or drug or alcohol abuse.

### Preoperative assessment

Baseline characteristics were collected 1 day before surgery, including patient demographic characteristics (e.g., sex, age, height, and weight), clinical characteristics (e.g., ASA grade, patient’s history of circulatory system, respiratory system, nervous system, and endocrine system), cognitive function status measured using the MMSE (19), and sleep quality measured using the Pittsburgh Sleep Quality Index (20). The age-adjusted Charlson comorbidity index (ACCI) scores (21) were calculated.

### Blood sample collection and biochemical analysis

We collected 2 ml of blood in a vacutainer (BD Biosciences, San Jose, CA, USA), which used EDTAK2 as an anticoagulant, after preoperative radial artery catheterization, and the blood sample was taken at 6:00 a.m. on the first day after surgery. The samples were centrifuged at 4,000 rpm/min for 10 min, and the supernatant was removed and used for detection or it was aliquoted and frozen −80^°^C until analysis.

The visfatin plasma concentration was measured using the enzyme immunoassay method (RayBiotech, Norcross, GA, USA) that had a lower limit of detection of 0.778 ng/ml. Albumin, creatinine, thyroid stimulating hormone (TSH), aspartate aminotransferase (AST), and alanine aminotransferase (ALT) levels were tested using a blood biochemical analyzer (HITACHI, Tokyo, Japan); white blood cells (WBCs), red blood cells (RBCs), platelets, and hemoglobin levels were tested using a blood analyzer (SYSMEX, Kobe, Japan). IL-1β and IL-6 levels were also tested using an immunosorbent assay (Boster, Wuhan, China).

### Anesthesia and analgesia

After admission, the geriatric doctor evaluated the pain of the patients before surgery. If the Numerical Rating Scale (NRS) score was greater than three, the patient would be given administrative drug Tylenine (containing oxycodone hydrochloride 5 mg and acetaminophen 325 mg) or Dolantin 50 mg according to their physical conditions.

Fascia iliaca block and subarachnoid spinal anesthesia was used. After entering the surgical theater, echocardiogram monitoring, invasive blood pressure through radial artery catheterization, and pulse oximetry monitoring were performed. All patients received ultrasound-guided fascia iliaca block before surgery and were administered 30 ml of 0.4% ropivacaine. For spinal anesthesia, the L2–3 or L3–4 levels was selected to puncture, and 8–10 mg of 0.3% ropivacaine was used, and no epidural catheter was placed.

All patients were treated with patient-controlled intravenous analgesia after surgery. The drug regimen was as follows: sufentanil 0.1 μg/kg, flurbiprofen axetil 200 mg, tropisetron hydrochloride 10 mg and normal saline to a volume of 100 ml. When the postoperative NRS pain scores was greater than three points or the patient actively requested additional analgesia because they were experiencing pain, the rescue analgesic drug Tylenine or Dolantin 50 mg was administered (22).

### Delirium assessment

The 3-min diagnostic interview for Confusion Assessment Method (3D-CAM) is an efficient and reliable way to determine whether a patient has delirium. The 3D-CAM assessment can be completed in an average of 3 min, and it has excellent performance compared with other evaluation methods. Its sensitivity and specificity are about 84.6–87.2% and 96.7–97.4%, respectively (23). A geriatrician trained by a professional psychiatrist performed 3D-CAM assessments twice daily (morning and afternoon) on patients during the first two postoperative days (24).

### Sample size

A piecewise regression model was used in the present study to examine the association between preoperative visfatin and POD. Five events per variable (EPV) is a widely used minimum criterion for sample size considerations in regression analysis. It was estimated that five variables could be included in the final model. For a given number of EPVs, 5 × EPV events are required in the analysis sample. For an EPV value of five, at least 25 events (development of POD) were required for the analysis. With an anticipated dropout rate of 10%, 28 POD patients were required. Additionally, a previous study showed that approximately 16% of patients who underwent hip replacement surgery developed POD, and thus, a minimum sample size of 176 was required for this study.

### Statistical analysis

Analysis was performed using SPSS v.25.0 (IBM Corp., Armonk, NY, USA) and R statistical software (version 4.2.0, R Core Team, Vienna, Austria). The intergroup comparison for normally distributed measurement data was performed using an independent sample *t*-test, and the results are presented as the mean and standard deviation (SD). Measurement data with a skewed distribution was presented as the median (quartile), and the intergroup comparison was performed using the Mann–Whitney U-test. Categorical variables were described using the frequency (%), and the intergroup comparisons were described using Pearson chi-square or Fisher’s exact probability test. Logistic regression was used for multivariate analysis. The correlation between visfatin and IL-6 is based on the Pearson or Spearman analysis.

Restricted cubic splines were used to analyze the association between preoperative plasma visfatin and the risk of POD. When there was evidence of non-linearity, a piecewise regression model was next fitted. The package segmented (version 1.4-1) was used to fit piecewise regression models, while adjusting for covariates. Mediation analysis was using mediation package (25).

## Results

### Patients characteristics

Among 176 patients who underwent hip fracture surgery, 18.2% (32 of 176) developed POD in our study population. The average age of patients in the POD group was 82.5 ± 7.4 years, which was significantly older than that of patients in the non-POD group (76.8 ± 7.9 years, *p* < 0.01). There were sixty-six (23.6%) and 10 (31.2%) men in the non-POD and POD groups, respectively, which was not significantly different (*p* > 0.05). Demographics such as gender, BMI, ASA, ADL, and education, did not show a group difference (*p* > 0.05). However, preoperative MMSE scores for patients in the POD group were significantly lower than those in the non-POD group (*p* < 0.01). Additionally, ACCI scores (*p* = 0.04) and postoperative plasma IL-6 levels (*p* = 0.002) were significantly higher in the POD group compared with that in the non-POD group. Other biochemical tests such as preoperative plasma glucose, creatinine, TSH, AST, ALT, WBC, RBC, and postoperative IL-1β levels showed no differences (*p* > 0.05). Additionally, preoperative plasma visfatin levels showed no difference between the two groups (Table 1).

**TABLE 1:** Associations between perioperative variables and postoperative delirium.

	Non-POD group (*n* = 144)	POD group (*n* = 32)	Statistical test (t/χ^2^/z)	*P*-value
**Preoperative data**				
Age (years)	76.8 ± 7.9	82.5 ± 7.4	–3.7	<0.001**
Sex (female)	110 (76.4%)	22 (68.8%)	0.8	0.367
BMI (kg/m^2^)	23.4 ± 3.8	23.2 ± 3.0	0.4	0.705
**ASA physical status class**			0.1	0.796
II	91 (63.2%)	21 (65.6%)		
III	53 (36.8%)	11 (34.4%)		
Education (years)	9.4 ± 4.1	9.3 ± 4.4	0.1	0.889
MMSE score (points)	25.5 ± 1.3	24.5 ± 1.0	4.9	<0.001**
ADL score (points)	92.9 ± 13.5	92.0 ± 10.5	0.4	0.718
PSQI score (points)	13.2 ± 5.7	15.2 ± 5.2	–1.8	0.077
ACCI score (points)	4.3 ± 1.4	4.9 ± 1.4	–2.1	0.040*
Admission–Operation time(hours)	32.5 (27.0, 45.0)	35.0 (27.0, 45.0)	–0.1	0.946
Ischemic heart disease	35 (24.3%)	8 (25.0%)	< 0.1	0.934
Chronic obstructive pulmonary disease	8 (5.6%)	1 (3.1%)	0.3	0.572
Hypertension	68 (47.2%)	21 (65.6%)	3.5	0.060
Diabetes	44 (30.6%)	8 (25.0%)	0.4	0.533
Stroke	29 (20.1%)	3 (9.4%)	2.0	0.153
Blood glucose (mmol/L)	8.6 ± 3.0	8.3 ± 2.7	0.7	0.504
Glycated hemoglobin (%)	6.5 ± 1.7	6.3 ± 1.2	0.4	0.670
Albumin (g/L)	40.4 ± 5.1	40.6 ± 3.3	–0.3	0.795
Creatinine (mmol/L)	68.0 ± 34.7	70.5 ± 28.5	–0.4	0.713
TSH (mIU/L)	2.8 ± 6.4	4.7 ± 16.1	–0.6	0.538
AST (IU/L)	22.9 ± 9.7	24.2 ± 9.3	–0.7	0.505
ALT (IU/L)	18.5 ± 8.6	21.6 ± 13.4	–1.2	0.230
WBC (×10^∧^9/L)	10.2 ± 3.2	11.1 ± 2.6	–1.6	0.111
RBC (×1012/L)	4.1 ± 0.6	4.1 ± 0.6	0.3	0.781
Platelet (×10^∧^9/L)	198.4 ± 53.5	202.8 ± 56.6	–0.4	0.685
Hemoglobin (g/L)	126.2 ± 16.7	125.1 ± 18.6	0.3	0.759
Visfatin (ng/ml)	43.2 (30.5, 70.7)	44.4 (20.5, 112.6)	–0.2	0.848
Preoperative plasma IL-1β (pg/ml)	26.5 (9.4, 59.1)	24.1 (1.8, 44.6)	–0.9	0.375
Preoperative plasma IL-6 (pg/ml)	9.5 (0.5, 22.7)	20.2 (0.1, 40.8)	–1.2	0.215
**Intraoperative data**				
Duration of surgery (min)	62.6 ± 26.4	55.2 ± 16.3	1.5	0.139
Duration of anesthesia (min)	88.0 ± 28.4	79.2 ± 17.7	1.7	0.100
Blood loss (ml)	200.0 (100.0, 300.0)	200.0 (100.0, 300.0)	–1.2	0.242
**Postoperative items**				
Postoperative serum IL-1β (pg/ml)	18.3 (1.4, 40.5)	13.6 (0.5, 30.7)	–1.0	0.315
Postoperative serum IL-6 (pg/ml)	101.5 (41.6, 186.3)	167.5 (121.1, 262.7)	–3.1	0.002
Hospitalization days	3.0 (3.0, 4.0)	3.0 (3.0, 4.0)	–0.7	0.480

Categorical variables are expressed as the *n* (%). Normally distributed data are presented as the mean ± SD, whereas, non-normally distributed data are expressed as the median (25th percentile, 75th percentile). BMI, body mass index; ASA, American Society of Anesthesiologists; MMSE, mini-mental state examination; ADL, activities of daily living; PSQI, Pittsburgh sleep quality index; ACCI, age-adjusted Charlson comorbidity index; TSH, thyroid stimulating hormone; AST, aspartate aminotransferase; ALT, alanine aminotransferase; WBC, white blood cell; RBC, red blood cell; IL-1β, interleukin-1β; IL-6, interleukin-6; SD, standard deviation. *p < 0.05, **p < 0.01.

Univariate logistic analysis showed that four factors, including age, preoperative MMSE scores, ACCI, and postoperative plasma IL-6, were statistically associated with POD in our research. Further multivariable analysis showed that three factors including age, low preoperative MMSE scores, and low postoperative plasma IL-6 levels were independent risk factors in POD among older hip fracture patients (Supplementary Table 1).

Among 32 POD patients, one patient had POD on the second day after operation (3.1%), and the other 31 patients had POD on the first day after operation (96.8%).

### Correlation between preoperative plasma visfatin and postoperative delirium

**FIGURE 2:**
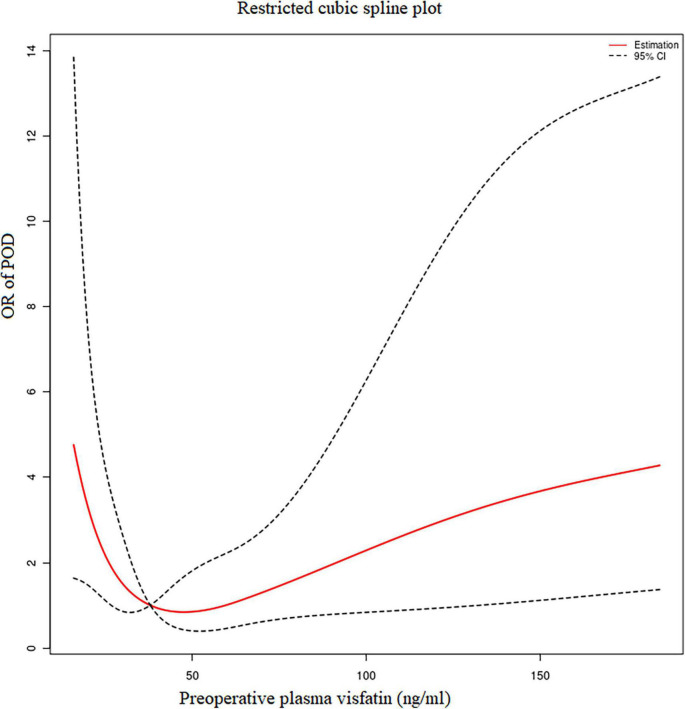
Restricted cubic spline analysis results. The relationship between the preoperative visfatin levels and OR of POD. OR, odd ratio; POD, postoperative delirium.

Using R 4.2.0 software, we found the preoperative plasma visfatin concentrations had a non-linear relationship with the risk of POD, with an inflection point between 30 and 50 ng/ml (Figure 2). When the preoperative visfatin level was lower than that of the inflection point, the risk of POD decreases as the preoperative visfatin level increases. Above this inflection point, the risk of POD increases with increasing preoperative visfatin levels.

### Break-point value by segmented regression

Segmented regression performed using R 4.2.0 software showed that the break-point value was 37.87 ng/ml. A decrease in the risk of POD was observed within the lower range until 37.87 ng/ml, with a hazard ratio of 0.59 per 5 ng/ml (OR = 0.59, 95% CI = 0.37–0.95), which increased thereafter (*P* for non-linearity < 0.001), with a hazard ratio of 1.116 per 10 ng/ml (OR = 1.10, 95% CI = 1.02–1.23).

### Pearson correlation analysis of the relationship between visfatin and inflammatory factors

**FIGURE 3:**
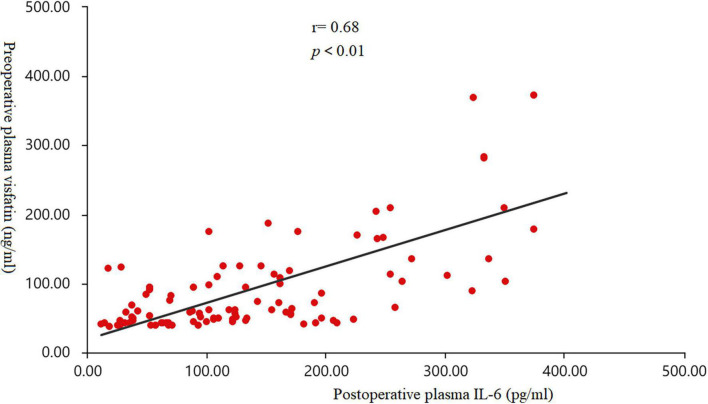
Scatter diagram. The correlation between the concentrations of preoperative plasma visfatin and postoperative plasma interleukin (IL-6) levels in patients with a plasma visfatin level of more than 37.87 ng/ml (r = 0.68, *p* < 0.01). IL-6, interleukin-6.

Pearson correlation analysis were used to analyze the correlation between visfatin and inflammatory factors. When the concentrations of preoperative plasma visfatin was lower than 37.87 ng/ml, there was no correlation between visfatin and IL-6 or IL-1 (Table 2), but when the preoperative plasma visfatin level was higher than 37.87 ng/ml, there was a positive correlation between concentrations of preoperative plasma visfatin and concentrations of postoperative plasma IL-6 (Table 3). Their relationship showed in a scatter plot (Figure 3).

**TABLE 2:** Pearson correlation analysis between visfatin and inflammation factors when visfatin concentrations was lower than 37.87 ng/ml.

		Preoperative plasma visfatin
Preoperative plasma IL-1β	Correlation coefficient	−0.10
	*P*-value	0.409
Preoperative plasma IL-6	Correlation coefficient	−0.10
	*P*-value	0.428
Postoperative plasma IL-1β	Correlation coefficient	−0.13
	*P*-value	0.323
Postoperative plasma IL-6	Correlation coefficient	0.03
	*P*-value	0.833

IL-1, interleukin-1; IL-6, interleukin-6.

**TABLE 3:** Pearson correlation analysis between visfatin and inflammation factors when visfatin concentrations was above 37.87 ng/ml.

		Preoperative plasma visfatin
Preoperative plasma IL-1β	Correlation coefficient	−0.12
	*P*-value	0.220
Preoperative plasma IL-6	Correlation coefficient	0.07
	*P*-value	0.470
Postoperative plasma IL-1β	Correlation coefficient	−0.12
	*P*-value	0.231
Postoperative plasma IL-6	Correlation coefficient	0.68**
	*P*-value	<0.001

IL-1, interleukin-1; IL-6, interleukin-6. *p < 0.05, **p < 0.01.

### Mediation analysis of the relationship between visfatin, interleukin-6, and postoperative delirium

**FIGURE 4:**
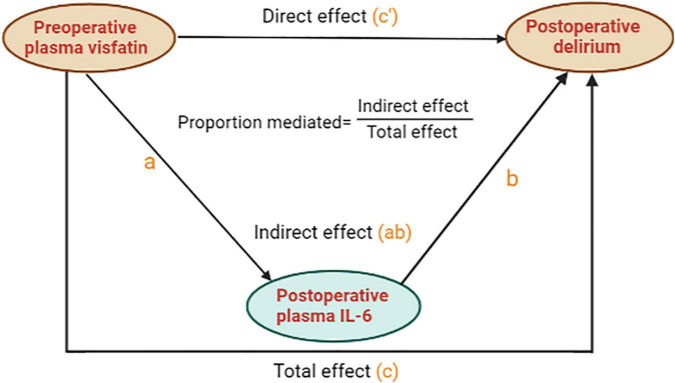
A mediation analysis model diagram. For brevity and clarity, a simplified version of the calculated model is presented. “c” means the total effect of preoperative plasma visfatin on postoperative delirium (POD) is represented by “c”, whereas, “c”’ means the direct effect of preoperative plasma visfatin on POD after controlling for the level of postoperative plasma interleukin (IL-6); “a” means the effect of preoperative plasma visfatin on the level of postoperative plasma IL-6; “b” means the effect of postoperative plasma IL-6 on POD, after controlling for preoperative plasma visfatin, the indirect effect of preoperative plasma visfatin on POD through postoperative plasma IL-6 can then be quantified as the product of “a” and “b”. IL-6, interleukin-6; POD, postoperative delirium.

We further analyzed whether preoperative plasma visfatin can affect the occurrence of POD by regulating inflammatory using mediation analysis. When the concentration of visfatin was higher than 37.87 ng/ml, the total effect of preoperative plasma visfatin on POD (c) could be divided into direct effect (c’) and indirect effect (a and b), the indirect effect was exerted by affecting postoperative plasma IL-6. Here, we use an easy model to show the relationship between them (Figure 4). The coefficients of total effect, direct effect and indirect effect were showed in Table 4.

**TABLE 4:** Coefficients of mediation analysis.

Variables	POD					Postoperative serum IL-6					POD				
	*B*	Standard error	*t*	β	*p*	*B*	Standard error	*t*	β	*p*	*B*	Standard error	*t*	β	*p*
Constant	1.204	1.064	1.131	–	**0.261**	363.744	221.126	1.645	–	**0.104**	0.757	1.05	0.721	–	**0.473**
Age	0.011*	0.005	2.106	0.245	**0.038**	0.388	1.084	0.358	0.034	**0.721**	0.011*	0.005	2.07	0.235	**0.041**
MMSE	−0.074*	0.033	–2.244	–0.243	**0.027**	–11.74	6.875	–1.708	–0.152	**0.091**	–0.06	0.033	-83	–0.196	**0.071**
PSQI	–0.002	0.006	–0.32	–0.03	**0.75**	–0.437	1.296	–0.337	–0.026	**0.737**	–0.001	0.006	-0.24	–0.022	**0.811**
ACCI	–0.041	0.029	–1.404	–0.164	**0.164**	–0.646	6.077	–0.106	–0.01	**0.916**	–0.04	0.028	−1.416	–0.161	**0.16**
Hypertension	0.05	0.076	0.656	0.066	**0.514**	–15.808	15.841	–0.998	–0.082	**0.321**	0.069	0.075	0.931	0.091	**0.354**
Preoperative plasma visfatin	0.001**	0	2.871	0.271	**0.005**	0.846**	0.1	8.439	0.654	**0.000**	0	0.001	0.55	0.068	**0.584**
Postoperative plasma IL-6											0.001*	0	2.476	0.31	**0.015**
*R* ^2^	0.222					0.475					0.273				
Adjust *R*^2^	0.17					0.439					0.215				

The mediation analysis of the relationship between visfatin, postoperative plasma IL-6 and POD. *B*, non standardized regression coefficient; *β*, standardized regression coefficient; *t*, B/Standard error. POD, postoperative delirium; IL-6, interleukin-6. *p < 0.05, **p < 0.01. Bold values represent the *p*-value.

## Discussion

The results of our study show that visfatin has a dual effect on POD, when the concentration of preoperative visfatin is higher than 37.87 ng/ml, it may play a role by regulating the release of IL-6.

This study showed that increasing age, a low MMSE score, a high ACCI score, and high postoperative plasma IL-6 levels were independent risk factors for PODs. When the concentration of preoperative plasma visfatin was lower than 37.87 ng/ml, it was a negatively correlated with the risk of POD; when concentration was exceeded, it was positively correlated with the risk of POD, which was associated with postoperative plasma IL-6 levels.

Postoperative delirium is a serious complication, which can potentially lead to longer hospital stays and even permanent disabilities. Additionally, POD may increase the risk of delirium-related dementia. Many scholars have conducted researches to prevent and reduce the probability of POD occurrence. Some of our results are consistent with those of previous studies. Some studies showed that advanced age and multiple comorbidities are risk factors for POD (3, 26).

For the relationship between IL-6 and POD, many studies suggested that preoperative higher preoperative IL-6 blood levels were associated with POD occurrence (7, 27–29). However, our study did not show a difference in the preoperative plasma IL-6 concentration between the two groups, but the plasma IL-6 concentration on day one after surgery in the POD group was significantly higher than that in the non-POD group. This result may have occurred because we took blood samples from the patients on the morning of surgery instead of blood samples when the patients were just admitted to the hospital. After admission, the patients took non-steroidal anti-inflammatory drugs and painkillers, which reduced the inflammation, resulting in a decrease in the IL-6 level.

Visfatin is nicotinamide phosphoribosyltransferase (NAMPT), which includes extracellular NAMPT (eNAMPT) and intracellular NAMPT (iNAMPT). iNAMP is mainly involved in glucose metabolism, and eNAMPT regulates inflammatory factor expression (30). The relationship between the visfatin level in blood and cognitive function remains controversial. Some studies showed that elevated blood visfatin levels are associated with a cognitive decline (12, 14, 31). However, some studies indicated that loss of visfatin may harm neurons and impair cognitive function (18). To date, there have no relevant studies on POD, and our study is the first to explore the relationship between visfatin and POD.

In this study, we did not find a correlation between visfatin and IL-6 when the preoperative plasma visfatin concentration was less than 37.87 ng/ml. It is worth exploring the effect of visfatin on POD when below 37.87 ng/ml.

Visfatin plays a crucial role in glucose metabolism. Visfatin activates its target cells by binding to the insulin receptor (IR) and exerts multiple insulin-mimetic effects, including enhanced glucose uptake and increased triglyceride synthesis (10, 32–34). A secondary analysis indicated that type 2 diabetes was associated with POD occurrence (35). An observational study showed that the average blood glucose and blood glucose variability within 48 h of POD in patients after acute aortic separation are significantly higher than those of non-POD patients (36), which indicated that blood glucose fluctuations may cause cognitive dysfunction in older patients. A high basal blood glucose level may cause direct neuron damage, and compared with long-term blood sugar fluctuations, short-term (acute) blood glucose fluctuations may cause more damage to neurons and lead to cognitive dysfunction (37). We hypothesized that visfatin effects at lower levels may occur through regulation of glucose metabolism, thereby affecting POD occurrence. Unfortunately, in this study, we did not record patients’ blood glucose level at multiple time points during the perioperative period. We will next study the relationship between visfatin and the fluctuation of blood glucose.

Our study showed that an elevated visfatin concentration in the blood above a certain level will promote IL-6 expression and increase the risk of POD occurrence. Visfatin has a pro-inflammatory effect. Numerous studies have shown that visfatin activates monocytes to produce pro-inflammatory cytokines (IL-1β, IL-6, and TNF-α) (38–40). However, in our study, we did not detect other inflammatory factors such as TNF-α or C-reactive protein in patients’ blood samples, so it is not possible to determine whether visfatin affects POD occurrence by regulating other inflammatory factors. We will detect them in our future studies.

The main advantages of this study are as follows: our study is the first to explore the relationship between visfatin and POD. Our study exhibits that novel inflammatory factors “visfatin” has a dual effection on the occurrence of POD, which provides ideas for follow-up research and may provide some guidance for POD prediction. Furthermore, our study shows when visfatin concentration was higher than 37.87 ng/ml, it may affect POD through the medication effect on postoperative plasma IL-6. We demonstrated that there is a correlational relationship between high pre-operative visfatin levels, elevated post-operative levels of IL-6 and POD.

The main limitations of this study are as follows: (1) This study is a single center study with a relatively small sample size. In future studies, we will conduct a multi-center study; (2) the numbers of kinds of inflammatory factors detected in this study was too limited so we cannot make it clear that whether visfatin affect POD through other inflammatory factors (3) this study did not detect the central visfatin, and this issue will be investigated in our follow-up studies. (4) The patients in this study were discharged on the third day after surgery or transferred to the rehabilitation department for further treatment, so this study only evaluated the complications of 2 days after surgery. The follow-up study will observe and follow up the long-term prognosis of patients.

## Conclusion

In summary, our study showed that there is a non-linear relationship between preoperative plasma visfatin and POD. Preoperative plasma visfatin has a dual effect on POD. Visfatin is the protective factor against POD when its preoperative plasma concentration is below 37.87 ng/ml. However, when it exceeds 37.87 ng/ml, the visfatin concentration becomes a risk factor for POD, which is mediated by postoperative plasma of IL-6.

## Data availability statement

The raw data supporting the conclusions of this article will be made available by the authors, without undue reservation.

## Ethics statement

The studies involving human participants were reviewed and approved by Beijing Jishuitan Hospital Medical Science Research Ethics Committee (JLKS202103-35). The patients/participants provided their written informed consent to participate in this study.

## Author contributions

NK, YY, NY, GW, and XG contributed to the study design. XG, YY, NY, and ZL obtained the funding. WZ, GW, and YY performed the anesthesia. NK and KZ collected the blood samples, contributed to the data collection, and responsible for data statistics. JH, YL, LC, and YL are laboratory technicians. YH, YS, and NK verified the underlying data. NK and YY drafted the manuscript. XG, KZ, YY, and NY reviewed the manuscript. All authors read and approved the final version of the manuscript.
