# Supplementary material for: Preoperative plasma visfatin may have a dual effect on the occurrence of postoperative delirium
Source: Front Med (Lausanne). 2022 Nov 22;9:1024942. doi: 10.3389/fmed.2022.1024942 (PMC9722954; doi:10.3389/fmed.2022.1024942)
Supplement: Supplementary file 1 [file Table_1.docx]

| Variables | Univariate analysis | | | Multivariable analysis | | |
| --- | --- | --- | --- | --- | --- | --- |
|  | OR value | OR value 95% CI | *p* value | OR value | OR value 95% CI | *p* value |
| Age | 1.095 | 1.039 ~ 1.153 | 0.001 | 1.084 | 1.017 ~ 1.155 | 0.013 |
| MMSE | 0.420 | 0.269 ~ 0.657 | 0.000 | 0.453 | 0.275 ~ 0.745 | 0.002 |
| ACCI | 1.301 | 1.006 ~ 1.682 | 0.045 | 0.959 | 0.678 ~ 1.358 | 0.815 |
| PSQI | 1.063 | 0.993 ~ 1.139 | 0.079 | 1.065 | 0.979 ~ 1.158 | 0.141 |
| Hypertension | 2.134 | 0.959 ~ 4.746 | 0.063 | 2.192 | 0.850 ~ 5.656 | 0.105 |
| Postoperative plasma IL-6 | 1.005 | 1.002 ~ 1.009 | 0.004 | 1.005 | 1.001 ~ 1.009 | 0.016 |

**Supplementary Table 1.** **Associations between perioperative variables and postoperative delirium**.

*Factors with *p*<0.10 in univariable analyses or were considered clinical important were included in multivariable logistic model. MMSE, Mini-Mental State Examination; PSQI, Pittsburgh Sleep Quality Index; ACCI, age-adjusted Charlson comorbidity index, IL-6, interleukin-6
